# The Preconditioning of Berberine Suppresses Hydrogen Peroxide-Induced Premature Senescence via Regulation of Sirtuin 1

**DOI:** 10.1155/2017/2391820

**Published:** 2017-07-02

**Authors:** Xiaofei Zhu, Haodi Yue, Xiaofang Guo, Jingyi Yang, Jingshuo Liu, Jiangtao Liu, Ruijie Wang, Wenjuan Zhu

**Affiliations:** ^1^Department of Clinical Immunology, Research Center for Immunology, School of Laboratory Medicine, Xinxiang Medical University, Xinxiang 453003, China; ^2^Henan Collaborative Innovation Center of Molecular Diagnosis and Laboratory Medicine, Xinxiang Medical University, Xinxiang 453003, China; ^3^Xinxiang Assegai Medical Laboratory Institute, Xinxiang Medical University, Xinxiang 453003, China; ^4^Department of Microbiology, School of Basic Medical Sciences, Xinxiang Medical University, Xinxiang 453003, China

## Abstract

With a long history of application in Chinese traditional medicine, berberine (BBR) was reported to exhibit healthspan-extending properties in some age-related diseases, such as type 2 diabetes and atherosclerosis. However, the antiaging mechanism of BBR is not completely clear. By means of hydrogen peroxide- (H_2_O_2_-) induced premature cellular senescence model, we found that a low-concentration preconditioning of BBR could resist premature senescence in human diploid fibroblasts (HDFs) measured by senescence-associated *β*-galactosidase (SA-*β*-gal), accompanied by a decrease in loss of mitochondrial membrane potential and production of intracellular reactive oxygen species (ROS). Moreover, the low-concentration preconditioning of BBR could make cells less susceptible to subsequent H_2_O_2_-induced cell cycle arrest and growth inhibition. Experimental results further showed that the low concentration of BBR could induce a slight increase of ROS and upregulate the expression level of sirtuin 1 (SIRT1), an important longevity regulator. H_2_O_2_-induced activation of checkpoint kinase 2 (Chk2) was significantly attenuated after the preconditioning of BBR. The present findings implied that the low-concentration preconditioning of BBR could have a mitohormetic effect against cellular senescence triggered by oxidative stress in some age-related diseases through the regulation of SIRT1.

## 1. Introduction

Since the “free radical theory” was proposed by Harman in 1956, it is gradually recognized that excessive ROS, a potent inducer of cellular senescence, are implicated in a variety of age-related diseases, including cardiovascular disease, type 2 diabetes and Alzheimer's disease [1, 2]. Mitochondria are not only a major resource of cellular ROS generation but also a sensitive target for oxidative damage. Mitochondria damage induced by ROS could be indicated by loss of electron transport, mitochondrial membrane potential, and ATP generation, which could suggest respiratory dysfunction that exacerbate ROS generation and lead to the “vicious cycle” [3]. As the major ROS within cells, H_2_O_2_ is regarded as a key source of hydroxyl radical modifying and mutating mitochondrial DNA (mtDNA), which was proved to be associated closely with premature aging. Experimental evidence also proved that exposure of young HDFs to subcytotoxic concentration of H_2_O_2_ accelerated cellular senescence, which displays several replicative senescent-like features including senescence-associated *β*-galactosidase (SA-*β*-gal) activity, an irreversible growth arrest in G1 and change in expression level of many genes [4–6].

Berberine (BBR) is a botanical alkaloid and the major bioactive compound in the Chinese's herb *Rhizoma coptidis*, which had been utilized to treat diabetes and infection for decades in traditional Chinese medicine [7, 8]. Recently, it was reported that BBR could exhibit a lifespan-prolonged function by influencing the conversion of tryptophan into kynurenine in *Drosophila* [9]. Meanwhile, BBR was also proposed as a potential antiaging agent in oxidative DNA damage and could affect mitoxantrone-induced cellular senescence [10, 11]. But the antisenescence mechanism of BBR is still not very clear. Previously, we observed that BBR was able to protect hepatocytes from H_2_O_2_-induced cellular apoptosis by SIRT1 [12], which is NAD^+^-dependent histone deacetylase, and plays an important role in the lifespan-extending effect of caloric restriction and antiaging as the longevity regulator [13, 14]. Indeed, excessive ROS, especially H_2_O_2_, could accelerate cellular senescence by affecting the function and expression level of SIRT1 [15, 16]. In this study, we wanted to confirm the hypothesis that BBR may be against H_2_O_2_-induced premature senescence by SIRT1 and explore the possible pharmacological mechanism of BBR involved with the regulation of SIRT1 in order to expand its potential application for age-related diseases.

## 2. Materials and Methods

### 2.1. Materials

Human fetal lung diploid fibroblast cell line 2BS was purchased from the Institute of Basic Medical Sciences, Chinese Academy of Medical Sciences. Berberine and dimethyl sulfoxide (DMSO) were obtained from Sigma-Aldrich Chemical Inc. (St. Louis, MO, USA). 3-(4,5-dimethyl-2-thiazolyl)-2,5-diphenyl-2H-tetrazolium bromide (MTT) was purchased from Promega (Beijing) Biotech Co., Ltd. ROS Detection Reagents H_2_DCFDA was from Molecular Probes (Molecular Probes, Eugene, OR, USA). Hydrogen peroxide was a product of Beijing Chemicals Reagent Co., Ltd (Beijing, China). Dulbecco's modified Eagle's medium (DMEM) with high glucose and fetal bovine serum were from Invitrogen (Carlsbad, CA, USA). Antibodies against Phospho-Chk2(#2661), Chk2(#3440), Sirt1(#2493), and HRP-linked anti-Rabbit IgG(#7074) and anti-Mouse IgG(#7076) were obtained from Cell Signaling Technology (Beverly, MA, USA), anti-actin antibody (sc-1616R) was from Santa Cruz Biotechnology (Santa Cruz, CA, USA), and ECL plus Western blotting kit was from GE Healthcare Bio-Sciences (Marlborough, MA, USA).

### 2.2. Cell Culture

The characters and application of human diploid fibroblasts for cellular senescence research have been reported [16]. Briefly, early passage 2BS in 20 population doublings (PDs) were cultured in DMEM supplemented with 10% fetal bovine serum, 100 IU/mL penicillin, and 100 *μ*g/mL streptomycin at 37°C in a humidified atmosphere of 5% CO_2_ and 95% air. According to different experiment requirements, 2BS cells were seeded into plates at an appropriate density. When confluent, 2BS cells were preincubated with the indicated concentrations of BBR for 12 hr before H_2_O_2_ was added to the medium. The H_2_O_2_ was freshly prepared from 30% stock solution and added to the culture medium at a final concentration of 200 *μ*M.

### 2.3. Cell Cycle Assay

20 PDs 2BS cells were seeded into 6-well plates at a density of 4 × 10^5^/well and cultured till confluence. After being synchronized by serum deprivation, cells were pretreated with or without BBR (12 *μ*M) for 12 hr under normal serum conditions and exposed to 200 *μ*M H_2_O_2_ for 2 hr. After that, cells were switched to fresh DMEM medium for 12 hr, digested by trypsin-EDTA, and collected by centrifuging at 1000 rpm for 5 min. Supernatant was discarded and cells were fixed in ice-cold 70% ethanol for 24 hr. At the time of analysis, cells were washed twice with PBS and then resuspended in 500 *μ*L PBS with 50 *μ*g/mL propidium iodide and 100 *μ*g/mL RNase A. After being incubated at 37°C for 30 min in the dark, DNA contents were analyzed for fluorescence with a BD FACSCalibur™ flow cytometer.

### 2.4. Senescence-Associated *β*-Galactosidase Staining

Cellular senescence was identified with *β*-galactosidase staining as described previously [6]. Briefly, after confluence, 2BS cells were pretreated with 6, 12, and 20 *μ*M BBR for 12 hr before a 2 hr exposure of 200 *μ*M H_2_O_2_. Next, cells were passaged at a ratio of 1 : 3 to 6-well plates with fresh DMEM medium for four days and then washed twice in PBS, fixed for 3~5 min with 3% formaldehyde, and washed with PBS again. After that, cells were incubated overnight at 37°C without CO_2_ in a freshly prepared staining buffer (1 mg/ml X-gal, 40 mM citric acid/sodium phosphate, pH 6.0, 5 mM potassium ferrocyanide, 5 mM potassium ferricyanide, 150 mM NaCl, and 2 mM MgCl_2_).

### 2.5. Intracellular Reactive Oxygen Species (ROS) Assay

20 PDs 2BS cells were cultured to reach confluence in 6-well plates and treated with 12 *μ*M BBR for 12 hr prior to a 2 hr exposure of 200 *μ*M H_2_O_2_, or only treated with BBR. Then, cells were stained with 10 *μ*M H_2_DCFDA for 30 min according to the manufacturer's manual. Oxidation of the probes to 2′,7′-dichlorofluorescein (DCF) was measured by BD FACSCalibur flow cytometry. Mean fluorescence intensity (MFI) was calculated and analyzed by CellQuest software. The percentage of ROS generation was calculated according to MFI value of DCF.

### 2.6. Measurement of Mitochondrial Membrane Potential (ΔΨm)

To study the ΔΨm changes, cells were stained with Rh123 at a final concentration of 10 *μ*g/mL for 30 min in the dark, washed with PBS twice, and then centrifuged at 500 × g for 10 min. Finally, fluorescence of Rh123 was immediately detected by BD FACSCalibur flow cytometry. Mean fluorescence intensity (MFI) was calculated and analyzed using CellQuest software. The percentage of mitochondrial membrane potential loss was calculated according to MFI value.

### 2.7. Western Blot

2BS cells were washed with ice-cold PBS and collected by scraping, immediately lysed in RIPA buffer (10 mM Tris-HCl pH 7.4, 1% Triton X-100, 1% sodium deoxycholate, 0.1% SDS, 160 mM NaCl, 5 mM EDTA, 50 mM NaF, 10 % glycerol, 1 mM Na_3_VO_4_, and 5 mM sodium pyrophosphate) supplemented with 1 *μ*g/mL aprotinin, 10 *μ*g/mL leupeptin, and 1 mmol/L phenylmethylsulfonyl fluoride. The protein concentration of extracts was determined by Bradford assay. According to the relative molecular weight of interest proteins, equal amounts of protein samples were subjected to 6–15% SDS-PAGE and transferred onto PVDF membranes. The membranes were incubated with various antibodies at 1 : 1000 dilutions. Finally, they were incubated with horseradish peroxidase-conjugated secondary antibodies (1 : 2000). Visualization was detected with ECL Plus Western blotting Detection System according to the manufacturer's recommendation.

### 2.8. Statistical Analysis

Data are expressed as mean ± SEM, and differences between two groups were assessed by the Student *t*-test. Differences between multiple groups were assessed by one-way ANOVA (Tukey's test). *p* < 0.05 was considered significant.

## 3. Results

### 3.1. Berberine Attenuate H_2_O_2_-Induced Premature Senescence

**Figure 1:**
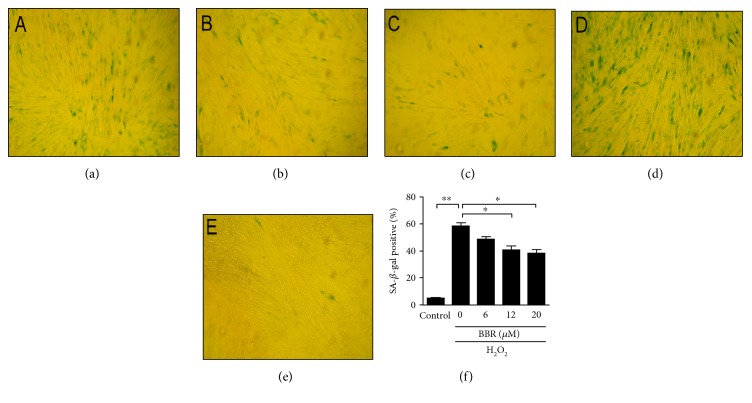
Antisenescence effect of BBR on H_2_O_2_-induced premature senescence in human diploid fibroblasts by *β*-galactosidase staining. 2BS cells were pretreated with different concentrations of BBR for 12 hr before exposure to 200 *μ*mol/L H_2_O_2_. For senescence assay, cells were cultured for four days with new fresh DMEM medium. On the fifth day, the blue precipitate can be seen by X-gal dye in senescent cells as the Materials and Methods mentioned. (a), (b), and (c) Pretreatment with 6, 12, and 20 *μ*mol/L BBR, respectively. (d) H_2_O_2_ treatment alone. (e) Untreated control. (f) The positive rates of SA-*β*-gal were calculated by counting the blue-dyed cells with a total of 200 cells at each visual field. ^∗^*p* < 0.05, ^∗∗^*p* < 0.01. The results are representative of three separate experiments.

**Figure 2:**
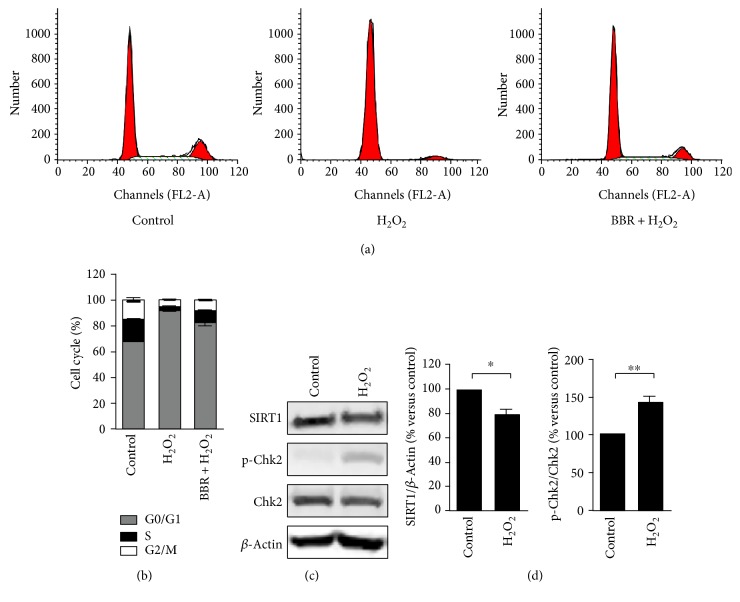
Cell cycle arrest and protein expression of H_2_O_2_-induced premature senescence in human diploid fibroblasts. 2BS cells were exposed to a sublethal concentration of 200 *μ*mol/L H_2_O_2_, or pretreated with 12 *μ*mol/L BBR for 12 hr before exposure. (a), (b) The influence of BBR in H_2_O_2_-induced cell cycle distribution. (c) The changes of SIRT1 expression and phosphorylation of Chk2 in H_2_O_2_-exposed human diploid fibroblasts. (d) Relative expression levels of Sirt1 and phospho-Chk2 in H_2_O_2_-exposed human diploid fibroblasts by gray analysis. ^∗^*p* < 0.05, ^∗∗^*p* < 0.01. The results are representative of three separate experiments.

Sublethal concentration H_2_O_2_ (<300 *μ*M) could cause growth arrest and promote cellular senescence but no adverse effect on human diploid fibroblasts survival [4, 6]. To investigate the effect of BBR on cellular senescence, we constructed the model of premature senescence via human fetal lung fibroblasts 2BS exposed to 200 *μ*M H_2_O_2_. As showed in Figure 1(d), H_2_O_2_ obviously induced premature cellular senescence indicated by a 58.6 ± 2.4% of SA-*β*-gal-positive cells compared with control group indicated by 4.8% ± 0.8% (Figure 1(e)). Meanwhile, H_2_O_2_ also induced significantly 92.2 ± 1.29% G1 phase arrest compared with 68.4 ± 2.5% of control (Figures 2(a) and 2(b)). However, the preconditioning of different concentrations of BBR (6–20) exhibited a significant reduction in the proportion of SA-*β*-gal-positive cells from 58.6 ± 2.4% to 48.8 ± 1.9%, 40.8 ± 3.0, and 38.1 ± 3.15, respectively (Figures 1(a), 1(b), 1(c), and 1(f)). In addition, the preconditioning of BBR at 12 *μ*M partially rescued H_2_O_2_-induced change of cell cycle from 92.2 ± 1.29% to 80.6 ± 2.5% in G1 phase, 2.2 ± 0.52% to 8.5 ± 1.1% in S phase, and 5.5 ± 0.78% to 8.8 ± 1.5% in G2/M phase (Figures 2(a) and 2(b)). Also, the preconditioning of BBR at 12 *μ*M significantly prevented H_2_O_2_-induced inhibition of cell growth from 41.8 ± 1.7% to 21.1 ± 4.5%. Interesting, when the concentration of BBR pretreated was 20 *μ*M, the cytoprotective effect of BBR was absent (Figure S1 available online at https://doi.org/10.1155/2017/2391820). These results suggested that low-concentration preconditioning of BBR had a beneficial effect on fibroblasts for resisting H_2_O_2_-induced cellular senescence.

### 3.2. Berberine Protect H_2_O_2_-Induced Damage of Mitochondrial Membrane

**Figure 3:**
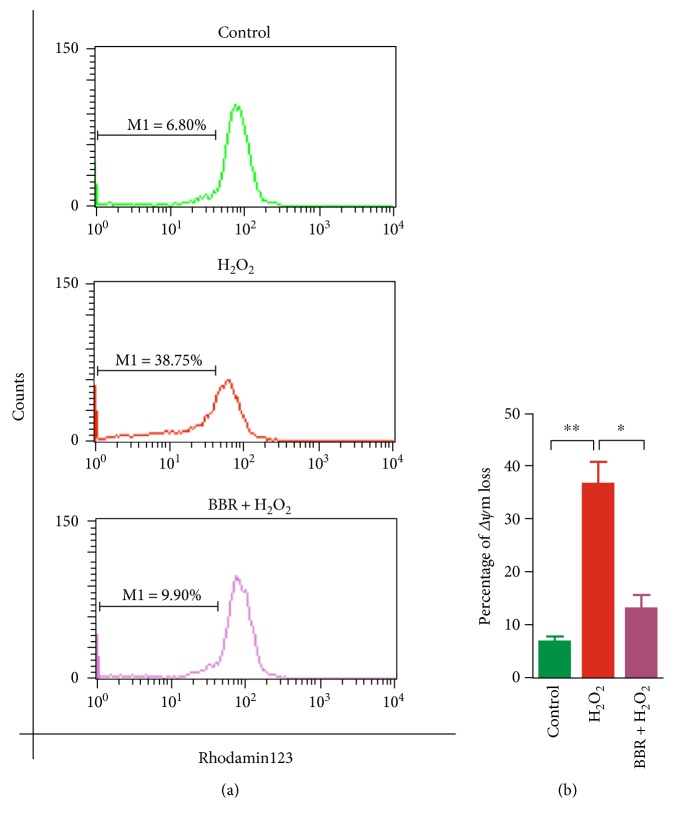
Protective effect of BBR on H_2_O_2_-induced mitochondrial membrane potential (Δ*ψ*m) loss in human diploid fibroblasts. 2BS cells were pretreated with or without 12 *μ*mol/L BBR for 12 hr before a 2 hr exposure to 200 *μ*mol/L H_2_O_2_ and then were stained with Rhodamin123. (a) The diagram from flow cytometry test. M1 represents the percentage of Δ*ψ*m loss. (b) Histogram of the mean fluorescence intensity of M1. ^∗^*p* < 0.05, ^∗∗^*p* < 0.01. The results are representative of three separate experiments.

**Figure 4:**
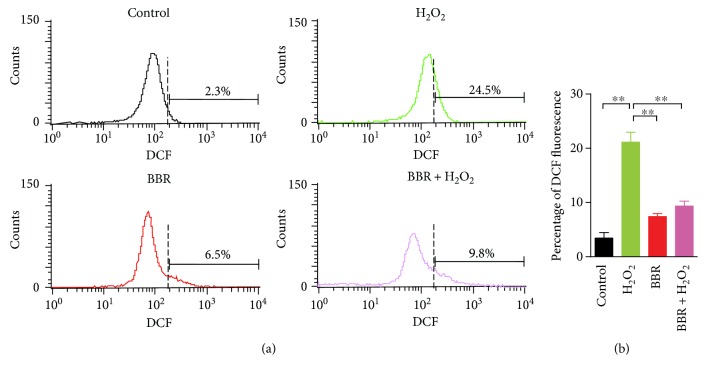
Protective effect of BBR on H_2_O_2_-induced intracellular ROS production in human diploid fibroblasts. 2BS cells were pretreated with or without 12 *μ*mol/L BBR prior to a 2 hr exposure of 200 *μ*mol/L H_2_O_2_, or treated with BBR alone as the Materials and Methods mentioned. (a) The diagram of fluorescence DCF detection with different treatments from flow cytometry analysis. (b) Histogram of the mean fluorescence intensity of DCF, reflecting the percentages of ROS generation in different treatment group. ^∗∗^*p* < 0.01. The results are representative of three separate experiments.

To examine the protective effect of BBR on mitochondrial membrane, Rhodamin123 was utilized for staining cells, which could selectively be retained in the mitochondria with an intact membrane potential, and be washed out of cells when mitochondrial membrane potential is lost [17, 18]. As shown in Figure 3, H_2_O_2_ obviously induced an increase of 2BS cells with ΔΨm loss from 6.7 ± 0.64% to 36.6 ± 4.5%. Indeed, pretreatment of BBR at 12 *μ*M decreased the percentage of 2BS cells with ΔΨm loss from 36.6 ± 4.5% to 13 ± 2.7%. Moreover, H_2_O_2_ exposure led to a significant increase to 21.1 ± 1.8% in intracellular ROS of 2BS cells by fluorescent probe DCF, compared with 3.4 ± 0.57% of untreated control. The percentage of intracellular ROS induced by H_2_O_2_ reduced obviously from 21.1 ± 1.8% to 9.4 ± 0.9% after the preconditioning of BBR at 12 *μ*M. Interestingly, BBR itself caused a slight increase in intracellular ROS generation (4 ± 1.6%), but nonsignificantly compared with untreated control (Figure 4). These data implied that the low-concentration preconditioning of BBR against H_2_O_2_-induced cellular senescence could be closely associated with BBR-induced mitochondrial stress, which subsequently plays an important role in the resistance of sublethal H_2_O_2_-induced damage.

### 3.3. Berberine Upregulates SIRT1 Expression and Reduces H_2_O_2_-Induced Chk2 Activation

**Figure 5:**
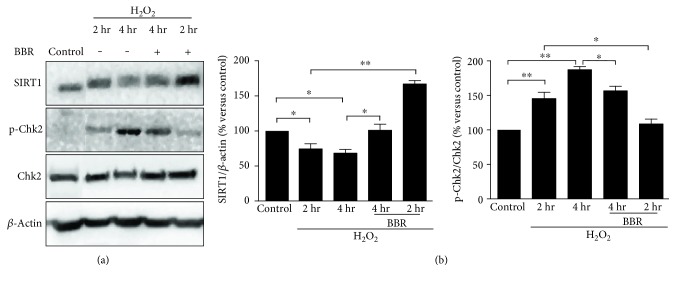
Protective effect of BBR on H_2_O_2_-induced reduction of SIRT1 expression and augment of phosphorylation of Chk2 in human diploid fibroblasts. 2BS cells were pretreated with 12 *μ*mol/L BBR for 12 hr before a 2 hr or 4 hr exposure to 200 *μ*mol/L H_2_O_2_. (a) Sirt1 and phospho-Chk2 was detected by western blotting. (b) Relative expression levels of Sirt1 and phospho-Chk2 in cells with different treatment by gray analysis. ^∗^*p* < 0.05, ^∗∗^*p* < 0.01. The results are representative of three separate experiments.

As our previous report, BBR could influence the survival of cells by an important longevity regulator SIRT1 [12]. Therefore, we examined the role of SIRT1 in H_2_O_2_-induced premature senescence of human diploid fibroblasts 2BS. As shown in Figure 2, H_2_O_2_ exposure for 2 hr induced a moderate decrease of SIRT1 expression in 2BS cells. When cells were exposed to H_2_O_2_ for 4 hr, the expression level of SIRT1 exhibited a significant reduction (Figure 5). It was interesting that BBR at 12 *μ*M alone could upregulate SIRT1 expression of fibroblasts 2BS in a time-dependent manner (Figure S2). H_2_O_2_ exposure also activated checkpoint kinase 2 significantly in a time-dependent manner (Figure 5), which is an event of the molecular signalling in H_2_O_2_-induced DNA damage response [19]. However, the preconditioning of BBR could not only protect from reduction of expression level of SIRT1 as same as did in our previous report, but also reduce phosphorylation of Chk2 (Figure 5). Meaningfully, the changes between SIRT1 expression and Chk2 phosphorylation by the preconditioning of BBR were inverse. These results hinted that the protective effect of low-concentration preconditioning of BBR on mitochondria or cellular senescence may be related to the upregulation of SIRT1 and deactivation of Chk2.

## 4. Discussion

Despite organismal complexity, the knowledge of basic aging or senescence mechanisms had been updated constantly for decades of research from cell-tissue culture to model organisms. Extending lifespan by biomedical interventions are scientifically plausible, such as calorie restriction and pharmacologically proaging pathway-targeted drugs [20, 21]. Here, we showed that a low concentration of BBR at 12 *μ*M could upregulate the expression level of SIRT1, an important longevity regulator, in human diploid fibroblasts, and the preconditioning of BBR at 12 *μ*M could suppress H_2_O_2_-induced cellular senescence by deactivating checkpoint kinase 2, which may subsequently prevent the downregulation of SIRT1.

Excessive ROS produced from the mitochondria is still regarded as one of the major factors for cumulative DNA damage to promoting aging. This mitochondrial free radical theory of cellular senescence is partly supported by the model of sublethal H_2_O_2_-induced replicative senescence in human diploid fibroblast [2, 3, 22]. Indeed, in our experiments, sublethal H_2_O_2_ increased significantly the number of senescent cells (Figures 1(d) and 1(f)), induced checkpoint kinase 2 activation (Figure 2(c)), and subsequently caused an irreversible growth arrest in G1 phase (Figures 2(a) and 2(b)). As a major and stable product formed by mitochondrial respiratory chain, excessive H_2_O_2_ can not only directly damage the integrity of DNA to cause genome instability but it can also be converted into hydroxyl radical, which injure mitochondrial membrane and lead to loss of Δ*Ψ*m. Consequently, loss of Δ*Ψ*m further can cause dysfunction of the mitochondrial electron transport chain and result in generation of more intracellular ROS [3, 22, 23]. Indeed, as shown in our experiments, H_2_O_2_ induced loss of Δ*Ψ*m (Figure 3()) and caused generation of more intracellular ROS (Figure 4()) in human diploid fibroblast. This suggested that mitochondrial dysfunction may be one of the reasons for acceleration of cellular senescence induced by sublethal H_2_O_2_.

In some studies, mitochondria-associated pathway can be manipulated to extend lifespan by pharmacological drugs, such as antioxidant and SIRT1-activtor resveratrol [24], antilipolytic acipimox [25], and antidiabetic metformin [26]. Berberine (BBR), the bioactive compound in the Chinese herb *Rhizoma coptidis*, had been reported as an antidiabetic drug with a long history in china [8, 27]. Recently, it also was found that BBR had an antiaging effect by AMPK-mediated inhibition of mTOR signaling [11]. However, AMPK activation by BBR may be attributed to its inhibition of mitochondrial respiratory electron transport chain, which causes a decrease of ATP production and leads to an increase of AMP to ATP ratio [28, 29]. AMPK activation also enhance SIRT1 activity by NAD^+^ levels [30]. Intriguingly, in our study, the expression level of SIRT1 was increased in a time-dependent manner when fibroblasts were treated at a low concentration (12 *μ*M) of BBR (Figure S2). Moreover, the preconditioning of BBR at 12 *μ*M obviously decreased the amount of senescent cells in a dose-dependent manner measured by the activity of senescence-associated *β*-galactosidase (Figures 1(a), 1(b), 1(c), and 1(f)), and partly reversed H_2_O_2_-induced G1 phase growth arrest (Figure 2(a)). These data suggested that upregulation of SIRT1 may be another antiaging mechanism of BBR. Actually, AMPK and SIRT1 are vital participators in cellular senescence by reciprocal regulation [29–32].

However, it was reported in many literature that BBR had a diphasic effect on different type of cells, such as protective effect on endothelial cells [33] and *β* cells [34] or apoptosis-induced effect on cancer cells [35, 36]. By MTT assay, we observed that BBR could protect cell from H_2_O_2_-induced growth inhibition at a low concentration (≤12 *μ*M), and at high concentration (≥20 *μ*M), BBR had no protective effect (Figure S1). Actually, this could be correlated with the concentration and intracellular distribution of BBR. BBR is a mitochondria-targeted cationic probe and can gather at the inner mitochondrial membrane by electrostatic interaction [37]. At a low concentration, BBR could mostly accumulate in the mitochondria and exhibit no cytotoxicity [38]. With an increase of concentration of BBR, the cellular localization of BBR distributed from the mitochondria to the cytoplasm and nuclei, which may inhibit cell growth and even induce cell death [39]. When targeted mitochondria are at low concentration, BBR could convert energy metabolism of cells into glycolysis by inhibition of mitochondrial respiratory chain [28, 38], which was indirectly reflected by a slight increase of intracellular ROS (Figure 4()). Recently, evidence indicated the beneficial role of mitochondrial ROS in lifespan at physiological or low concentration under stress conditions [40, 41]. It implied that low concentration of BBR may alert the mitochondria to trigger stress-mediated SIRT1 expression by an appropriate amount increase of intracellular ROS, and benefit fibroblasts to resist subsequent sublethal H_2_O_2_-induced stress.

SIRT1 is a master metabolic sensor and a mitochondria function protector by regulating some elements of proaging pathways, and delays the onset of age-related diseases [13, 14, 42–45]. Under oxidative stress, SIRT1 could exhibit antioxidative activity by upregulating expression of antioxidant enzymes [46, 47]. However, H_2_O_2_ could downregulate the expression level of SIRT1 by Chk2-HuR-mediated instability of SIRT1 mRNA [16]. Indeed, in our study, exposure to sublethal H_2_O_2_ induced mild downregulation of SIRT1 and Chk2 activation (Figures 2(c), 2(d), and 5(a)). But the H_2_O_2_-induced Chk2 activation was reduced significantly by pretreatment of BBR, accompanied by an obviously retard in downregulation of SIRT1 expression (Figure 5). These data implied that low concentration of BBR may also protect from reduction of SIRT1 expression by deactivating Chk2, which may be associated with BBR-induced upregulation of SIRT1.

Mitohormesis could be triggered by any insults including pharmacological intervention and induce the cellular stress response, and thereby protect against larger subsequent stresses [48]. More importantly, it was proposed that mitohormesis is a reason for benefits of ROS produced by lifespan-promoting interventions in cellular physiology under stress [49]. Recently, metformin, an antidiabetic drug, shows lifespan-extending properties by peroxiredoxin PRDX-2 related to ROS-mediated mitohormetic signalling pathway [26]. Based on our data, we speculate that the mitohormetic effect of BBR may be associated with the upregulation of SIRT1, which possibly is associated with a BBR-induced appropriate amount of increase of intracellular ROS. However, what is the mitochondrial retrograde signalling that induces SIRT1 expression during mitohormesis? Is ROS produced by low concentration of BBR really involved in mitohormesis as a signalling molecular? All of these questions will need to be answered by further deliberate experiments.

## 5. Conclusions

Our work revealed that a low-concentration preconditioning of BBR may exhibit antisenescence effect on H_2_O_2_-induced premature senescence of human diploid fibroblasts through the process of mitohormesis involved with SIRT1 upregulation, which elicits cytoprotective responses to resist subsequent H_2_O_2_-induced stress by decreasing activation of Chk2. In turn, this could protect against reduction of SIRT1 expression. This could give a hint that mitochondrial-targeted drugs like BBR can be potentially applied for aging-related diseases through mitohormesis.

## Supplementary Material

Figure. S1 Protective effect of BBR on H2O2-induced growth inhibition in human diploid fibroblasts. ∗ p<0.05, ∗ ∗ p<0.01.The results are representative of three separate experiments. Figure. S2 the expression level of SIRT1 in low concentration BBR-treated human diploid fibroblasts. 2BS cells were treated with 12μmol/L BBR for indicated time, then total protein was collected and detected SIRT1 by Western Blotting A: expression of SIRT1 in a time-dependent manner. B: Relative expression levels of Sirt1 by gray analysis. ∗ p<0.05, ∗ ∗ p<0.01.The results are representative of three separate experiments.
