# Supplementary figures and images for: The Preconditioning of Berberine Suppresses Hydrogen Peroxide-Induced Premature Senescence via Regulation of Sirtuin 1
Source: Oxid Med Cell Longev. 2017 Jul 2;2017:2391820. doi: 10.1155/2017/2391820 (PMC5511663; doi:10.1155/2017/2391820)

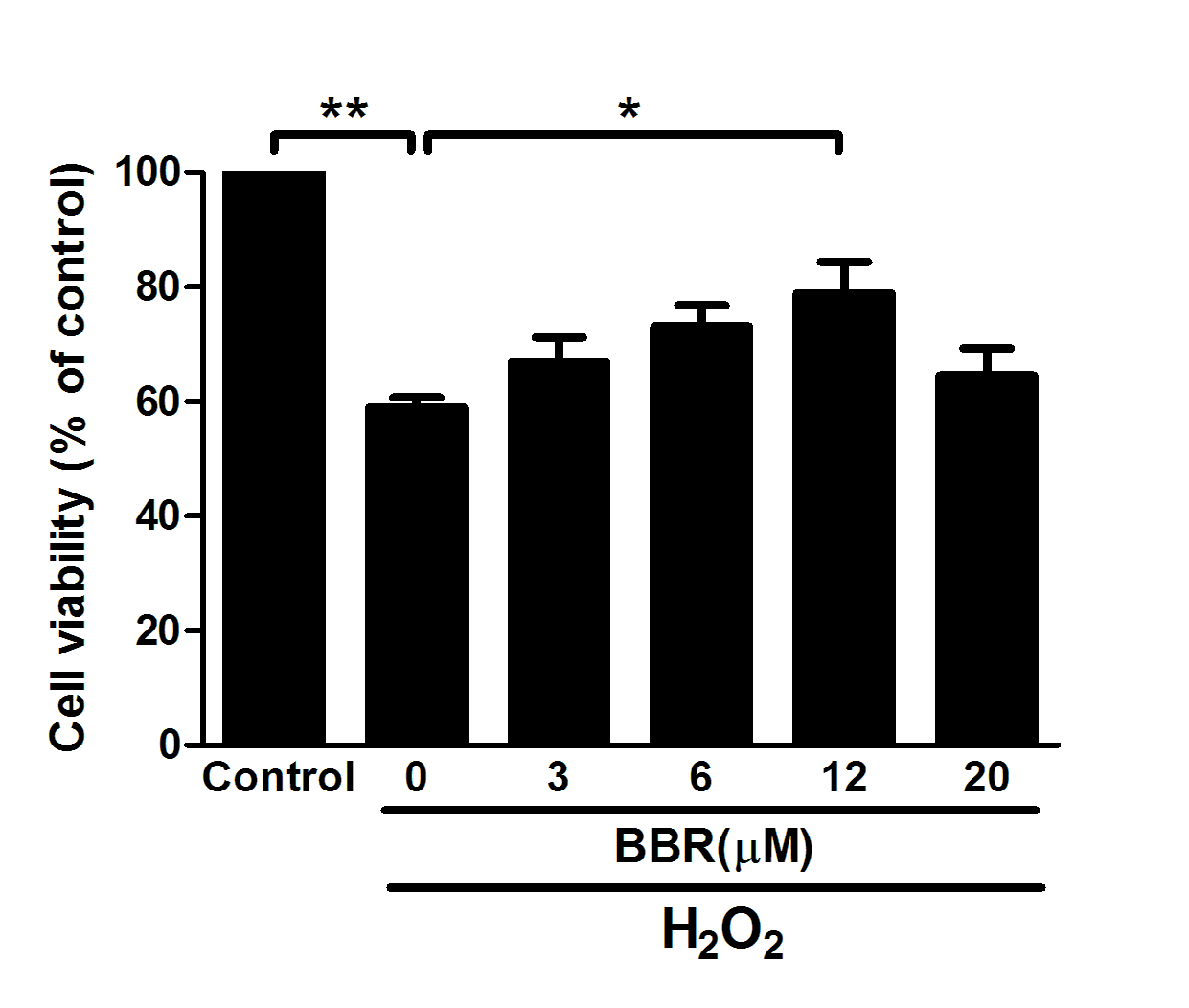

Supplement: Supplementary file 1 — Figure. S1 Protective effect of BBR on H2O2-induced growth inhibition in human diploid fibroblasts. ∗ p<0.05, ∗ ∗ p<0.01.The results are representative of three separate experiments. Figure. S2 the expression level of SIRT1 in low concentration BBR-treated human diploid fibroblasts. 2BS cells were treated with 12μmol/L BBR for indicated time, then total protein was collected and detected SIRT1 by Western Blotting A: expression of SIRT1 in a time-dependent manner. B: Relative expression levels of Sirt1 by gray analysis. ∗ p<0.05, ∗ ∗ p<0.01.The results are representative of three separate experiments. [file 2391820.f1.tif]

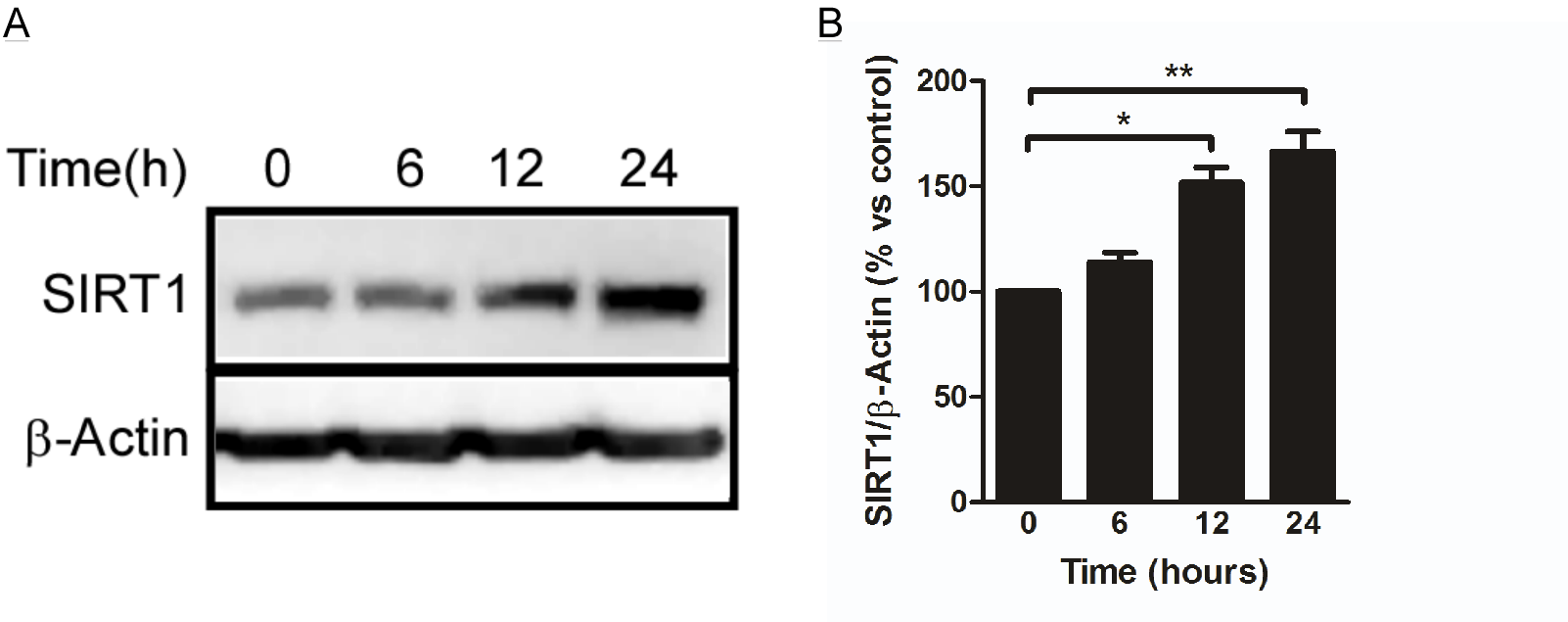

Supplement: Supplementary file 2 [file 2391820.f2.tif]
