# Supplementary material for: The Preconditioning of Berberine Suppresses Hydrogen Peroxide-Induced Premature Senescence via Regulation of Sirtuin 1
Source: Oxid Med Cell Longev. 2017 Jul 2;2017:2391820. doi: 10.1155/2017/2391820 (PMC5511663; doi:10.1155/2017/2391820)
Supplement: Supplementary file 4 [file 2391820.f4.pdf]

## Supplemental Experimental Procedures

***Cell viability assay.***

20 PDs 2BS cells were seeded into 96-well plates at a density of $4\times10^4$/well, and treated with different concentrations of BBR (0-20μM) for 12hr before a 2hr exposure of 200μM $H_2O_2$. After 48hr culture in fresh DMEM medium, cells were stained with MTT (10μg/ml) in PBS for 4hr, and then dissolved with dimethyl sulfoxide (DMSO). Plates were shaken for 10min and the absorbance was measured using a Multiskan MS Plate Reader (MTX Lab Systems, Inc. Virginia, USA) at 570nm. The rate of cell viability was calculated as followed: (absorbance of treated group－absorbance of blanks)/(absorbance of control－absorbance of blanks)×100 as a percentage.

## Supplemental Data

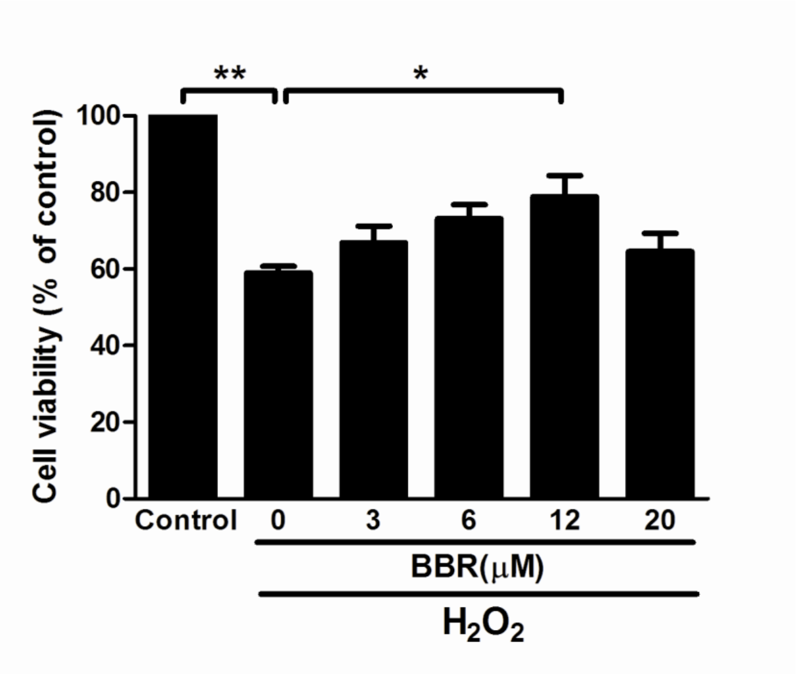


**Figure. S1 Protective effect of BBR on $H_2O_2$-induced growth inhibition in human diploid fibroblasts.** *p<0.05, **p<0.01.The results are representative of three separate experiments.

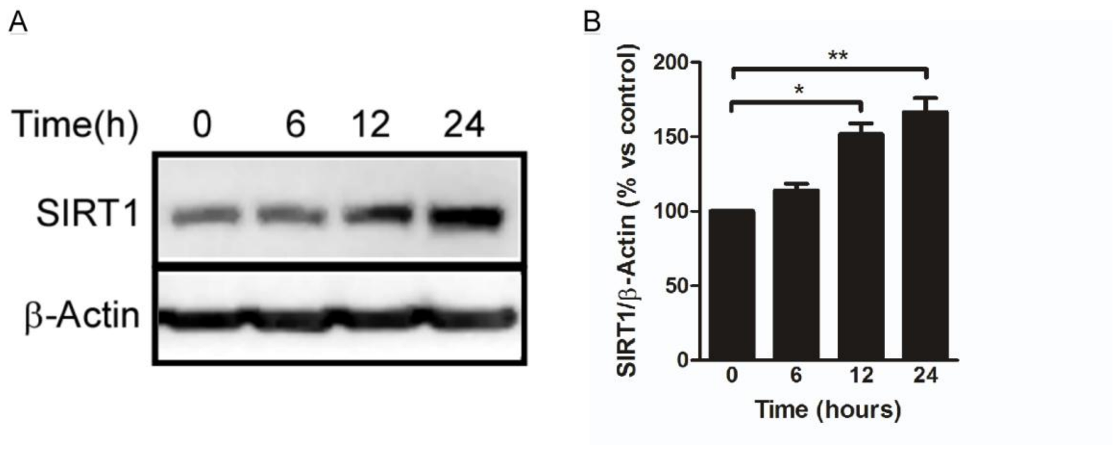


**Figure. S2 the expression level of SIRT1 in low concentration BBR-treated human diploid fibroblasts.**

2BS cells were treated with 12μmol/L BBR for indicated time, then total protein was collected and detected SIRT1 by Western Blotting **A:** expression of SIRT1 in a time-dependent manner. **B:** Relative expression levels of Sirt1 by gray analysis. *$p<0.05$, **$p<0.01$.The results are representative of three separate experiments.
